# A comprehensive review of the preclinical efficacy profile of the ErbB family blocker afatinib in cancer

**DOI:** 10.1007/s00210-014-0967-3

**Published:** 2014-03-19

**Authors:** Helmout Modjtahedi, Byoung Chul Cho, Martin C. Michel, Flavio Solca

**Affiliations:** 1 School of Life Science, Faculty of Science, Engineering and Computing, Kingston University London, Kingston upon Thames, UK; 2 Division of Medical Oncology, Yonsei University College of Medicine, Seoul, Republic of Korea; 3 Department of Pharmacology, Johannes Gutenberg University, Mainz, Germany; 4 Department of Regional Medicine and Scientific Affairs, Boehringer Ingelheim Pharma GmbH & Co. KG, Ingelheim, Germany; 5 Department of Pharmacology, Boehringer Ingelheim RCV GmbH & Co. KG, Doktor-Böhringer Gasse 5-11, 1120 Vienna, Austria

**Keywords:** Afatinib, Epidermal growth factor receptor, Non-small cell lung cancer, Resistance, Combination treatment

## Abstract

Afatinib (also known as BIBW 2992) has recently been approved in several countries for the treatment of a distinct type of epidermal growth factor receptor (EGFR)-mutated non-small cell lung cancer. This manuscript comprehensively reviews the preclinical data on afatinib, an irreversible inhibitor of the tyrosine kinase activity of members of the epidermal growth factor receptor family (ErbB) including EGFR, HER2 and ErbB4. Afatinib covalently binds to cysteine 797 of the EGFR and the corresponding cysteines 805 and 803 in HER2 and ErbB4, respectively. Such covalent binding irreversibly inhibits the tyrosine kinase activity of these receptors, resulting in reduced auto- and transphosphorylation within the ErbB dimers and inhibition of important steps in the signal transduction of all ErbB receptor family members. Afatinib inhibits cellular growth and induces apoptosis in a wide range of cells representative for non-small cell lung cancer, breast cancer, pancreatic cancer, colorectal cancer, head and neck squamous cell cancer and several other cancer types exhibiting abnormalities of the ErbB network. This translates into tumour shrinkage in a variety of in vivo rodent models of such cancers. Afatinib retains inhibitory effects on signal transduction and in vitro and in vivo cancer cell growth in tumours resistant to reversible EGFR inhibitors, such as those exhibiting the T790M mutations. Several combination treatments have been explored to prevent and/or overcome development of resistance to afatinib, the most promising being those with EGFR- or HER2-targeted antibodies, other tyrosine kinase inhibitors or inhibitors of downstream signalling molecules.

## Introduction

Epidermal growth factor (EGF), first described in 1962 (Cohen 1962), is a 53 amino acid peptide (Savage et al. 1972) which serves as an auto- and/or paracrine stimulator of cell growth, proliferation and differentiation. Its discovery was awarded in 1986 with the Nobel Prize in Physiology and Medicine to Stanley Cohen and Rita Levi-Montalcini. The receptor for EGF is called epidermal growth factor receptor (EGFR) and has been found overexpressed in many types of cancer (Modjtahedi and Dean 1994), where it mainly promotes proliferation and survival of malignant cells and, by inducing expression of angiogenic growth factors and metalloproteinases, promotes tumour vascularization and metastasis (De Luca et al. 2008). The identification of EGF and its receptor resulted in the discovery of three other members of the EGFR (also called HER or ErbB) family and their cognate ligands. These in turn led to the development of several therapeutic strategies against these receptors for use in the targeted therapy of human cancers (Ioannou et al. 2012; Zhang et al. 2007).

### Structure and function of ErbB family members

EGF affects cell function by binding to specific cell surface receptors which are part of the ErbB family (Holbro and Hynes 2004). Besides EGF, endogenous ErbB ligands include amphiregulin, transforming growth factor-α (TGF-α), epigen, epiregulin, heparin-binding EGF-like growth factor, neuregulin 1-4, neuroglycan, tomoregulin and betacellulin. The ErbB receptor family contains four closely related members, which are termed EGFR^1^ (also known as ErbB1 or HER1), HER2 (also known as ErbB2 or neu), ErbB3 (also known as HER3) and ErbB4 (also known as HER4) (Ioannou et al. 2012). ErbB family members are characterized by an extracellular ligand-binding domain, a transmembrane region and an intracellular domain with intrinsic tyrosine kinase activity. The crystal structures of the kinase domain of the EGFR have been reported (Kumar et al. 2008), including those with G719S, T790M and L858R mutations (Yasuda et al. 2012). The three-dimensional structures of the extracellular domain of some ErbB members have also been determined (Burgess et al. 2003) and revealed some insight on how this family of receptors gets activated and transduces extracellular signals to the cell interior.

Key to signal transduction is the mandatory formation of ErbB homo- or heterodimers. Upon agonist binding, EGFR, ErbB3 and ErbB4 undergo a conformational change which exposes sites for receptor dimerization. In general, ligand-induced ErbB receptor ectodomain dimerization triggers the formation of intracellular asymmetric kinase dimers in which the C-lobe of the activating monomer engages the N-lobe of the acceptor monomer (Zhang et al. 2006). In such dimers, the activating monomer acts as an allosteric activator by pushing the C-helix in the correct position for catalysis. Molecular promiscuity of the ErbB kinase domains results in transphosphorylation of C-terminal regulatory tyrosine residues in the intracellular domain of the activating kinase which acts as a substrate for the acceptor monomer. These phosphotyrosines become attachment sites for downstream signalling molecules, hence transducing signals from the cell surface to the nucleus via the Ras/extracellular signal-regulated kinase (ERK) pathway, the phosphatidyl-inositol-3-kinase (PI3K)/Akt pathway and signal transducers and activators of transcription (STAT) pathways (Kumar et al. 2008).

Interestingly, the HER2 ectodomain, for which a ligand has never been identified, is poised in an “active like” conformation. Therefore, HER2 is believed to act as the preferred dimerization partner for other ErbB family members (Graus-Porta et al. 1997).

ErbB3 on the other hand has three cognate ligands (neuregulin 1 and 2 and neuroglycan) but is distinct from other ErbB family members with respect to weak intrinsic tyrosine kinase activity due to structural diversity at critical catalytic residues in the kinase domain (Shi et al. 2010). Accordingly, ErbB3 works as a signalling entity only after heterodimerization with other ErbB receptor molecules (Holbro and Hynes 2004).

ErbB4, the fourth member in this family, exhibits complex signal transduction modalities. This receptor molecule can be expressed in four functionally variant isoforms (Hollmen and Elenius 2010). Two isoforms differ in the extracellular juxtamembrane domain (JM-a and JM-b) and two in the intracellular domain (CYT-1 and CYT-2). The CYT-1 isoform for instance contains a 16 amino acid insert exhibiting several binding sites for PI3K, while the CYT-2 isoform cannot couple to the PI3K pathway because it lacks the necessary YPTM binding sites. The JM-a and JM-b isoforms are also alternative splice variants. When compared to the three other variants, the longer JM-a molecule differs significantly in its mode of signal transduction and is characterized by the presence of specific residues that can be cleaved by the TNFα converting enzyme TACE. As a result, the ectodomain of ErbB4 is being shed and the truncated membrane-bound intracellular domain of ErbB4 (m80) becomes a substrate of γ-secretase. These two sequential proteolytic steps generate an intracellular ErbB4 variant known as s80 that translocates to the nucleus and acts as a transcriptional co-activator and co-repressor by interacting with transcription factors such as STAT5, YAP, ER, ETO2 or TAB2-NcoR. The large plasticity of the ErbB receptor system, which involves at least 13 different ErbB ligands and at least four different receptors excluding splice variants, results in a sophisticated signalling network with output control at multiple levels (Roskoski 2014; Yarden and Sliwkowski 2001).

### The importance of ErbB family members in cancer biology

ErbB receptors are expressed in many cell types of epithelial, mesenchymal and neuronal origin (Zhang et al. 2007). They exhibit aberrant signal transduction in many types of solid human cancers, including non-small cell lung cancer (NSCLC), breast cancer (BC), bladder cancer, ovarian cancer, colorectal cancer, pancreatic cancer and head and neck squamous cell cancer (HNSCC) (Ciardiello and Tortora 2008; Ioannou et al. 2012; Khelwatty et al. 2013). This exaggerated signalling may involve an enhanced signal input due to increased exposure to ErbB ligands (Modjtahedi and Dean 1994; Zhang et al. 2007). Secondly, tumour cells can overexpress ErbB receptors, due for instance to an increased gene copy number (Ciardiello and Tortora 2008) or defective down-regulation (Pareja et al. 2012). Thirdly, cancer cells may harbour mutations in EGFR, HER2 or ErbB4 (see below). The ErbB family members are involved not only in cell proliferation but also several other processes regulating tumour progression, such as cell motility, cell adhesion and angiogenesis (Zhang et al. 2007). Hence, overexpression of EGF receptors or deregulation of their activity is typically associated with the degree of malignancy of the disease and a poor prognosis.

HER2 was the first member in the ErbB family to be associated with epithelial cancers. The HER2 protein appears overexpressed in a variety of carcinomas including but not limited to BC, NSCLC and ovarian, gastric and salivary gland cancer (Heiser et al. 2012). ERBB2 gene amplification is driving protein overexpression in 18–25 % of BC cases. BC overexpressing HER2 are clinically more aggressive than those exhibiting normal numbers of HER2 protein at the cell surface (Press et al. 1990). While less abundant, cancer driving HER2 mutations have also been described in NSCLC (Shigematsu et al. 2005) and breast cancer (Bose et al. 2013).

EGFR mutations have been characterized best in NSCLC, where they represent early genetic events in cancer development, which are of fundamental relevance for the biology of these tumours (Gazdar 2009; Hammerman et al. 2009). Activating EGFR mutations are found in the ectodomain as well as in the ATP-binding pocket of the tyrosine kinase domain and led to aberrant activation. Kinase domain mutations are grouped into three classes (Gazdar 2009). Class I mutations are in-frame deletions within exon 19, almost always including a loss of amino acid residues 747–749 and accounting for 40 % of all EGFR mutations. Class II mutations are non-synonymous single nucleotide substitutions, most often replacing a leucine for arginine in codon 858 (L858R); other class II mutations exist (e.g. G719X) but are much less common. Class III mutations are in-frame duplications or insertions in exon 20. Interestingly, EGFR mutations are typically exclusive with KRAS and BRAF mutations, at least in lung cancer, and tumours exhibiting one of the latter two are relatively insensitive to EGFR inhibitors (Lin and Bivona 2012).

Oncogenic mutants also exist in other ErbB family members, but occur less frequently. For HER2, they mostly represent in-frame duplications or insertions in an eight-codon region of exon 20. Transgenic mice with inducible expression of the most common HER2 mutation, an in-frame YVMA insertion at residue M774, in mouse lung epithelium exhibit invasive adenosquamous cancer restricted to proximal and distal bronchioles (Perera et al. 2009). ErbB3 mutations have recently been reported in ~11 % of colon and gastric cancers (Jaiswal et al. 2013). Interestingly, the oncogenic activity of the ErbB3 mutants remains dependent on kinase active ErbB2 and was found to be effectively blocked by ErbB targeting antibodies or small molecule inhibitors. The majority of the mutations identified clustered in the extracellular domain, although some were found to map to the intracellular kinase domain. ErbB4 mutations have been described to occur in lung, breast, gastric cancer and melanoma and like for ErbB3 differ from those observed in EGFR or HER2 by not displaying characteristic mutational hotspots. Several of these ErbB4 mutations were shown to be oncogenic in melanoma models (Prickett et al. 2009).

### ErbB receptor inhibitors

Due to the importance of the ErbB family of receptors in tumour progression and poor response to the conventional therapies, intensive efforts have been devoted to identify and develop inhibitors for ErbB receptors (see Table 1).

**Table 1:** Potency of afatinib and other compounds to inhibit tyrosine kinase activity of ErbB family members including important mutations of EGFR. Data are shown as the range of the nanomolar concentration causing 50 % inhibition and are adapted from Solca et al. (2012)

	EGFR^WT^	EGFR^L858R^	EGFR^L858R/T790M^	HER2	ErbB4
Afatinib	0.2–0.7	0.2–0.4	9–10	7–25	0.7–1.7
Canertinib	0.3–1.7	0.4–0.8	18–36	22–72	0.8–10
Erlotinib	0.9–1.7	1.1–2.7	1,520–3,562	238–698	579–756
Gefitinib	0.4–4.7	0.8–1.4	534–1,267	416–1,830	293–323
Lapatinib	0.3–17	2–8	>4,000	6–25	18–30

One approach has focused on monoclonal antibodies against ErbB family members which interact with the extracellular domains. These included cetuximab (Horn and Sandler 2009) and panitumumab, targeting EGFR, as well as trastuzumab (Wong and Lee 2012) and pertuzumab, which target HER2. Originally, multiple anti-HER2 therapies ranging from therapeutic antibodies (Park et al. 1992) to antibody drug conjugates (Boyraz et al. 2013), small molecule inhibitors such as lapatinib (Hurvitz and Kakkar 2012) and liposomal vectors containing the E1A tumour suppressor known to repress the expression of HER2 as gene therapy option (Yoo et al. 2001) were simultaneously developed. The data from five clinical trials using trastuzumab in BC patients whose tumours overexpress HER2 ultimately led to the registration of trastuzumab in 1998 as the first anti-ErbB-targeted agent which has become nowadays the mainstay of therapy in HER2-positive BC (Perez et al. 2011).

Another approach was based on small molecules which directly bind to the tyrosine kinase domain of ErbB receptors and act as tyrosine kinase inhibitors (TKIs). The first representatives of this class, gefitinib, erlotinib and lapatinib, were reversible EGFR or EGFR/HER2 selective TKI inhibitors (Horn and Sandler 2009). These agents are initially effective in many patients but resistance may develop upon prolonged treatment. Irreversible ErbB family TKIs were developed in the hope that they may provide superior and more sustained efficacy as they inhibit all oncogenic homo- and heterodimers (Barf and Kaptein 2012).

In general, tumours which are not driven by a particular ErbB deregulation are not sensitive to monotherapy with ErbB targeting agents. In NSCLC patients with tumours displaying EGFR mutations, EGFR-TKIs have proven to be a very effective treatment option. Some rare EGFR mutations (e.g. class III exon 20 insertions), which are known to be activating, unfortunately show poor sensitivity to erlotinib or gefitinib and are one cause for primary resistance to these agents in EGFR mutant NSCLC patients (Vadakara and Borghaei 2012).

While gefitinib and erlotinib are initially effective in the majority of NSCLC patients with common EGFR mutations (exon 19 deletions and L858R point mutations), acquisition of resistance invariably occurs. Secondary or acquired resistance is the major limitation to the long-term use of ErbB TKIs. The two major mechanisms for the development of resistance during treatment are the occurrence of tumour cell clones which carry additional EGFR mutations or additional genetic alterations that can co-occur with EGFR-activating mutations (Lin and Bivona 2012). The latter include amongst others mutations in key molecular switches that control the activity of the PI3K/Akt pathway, insulin-like growth factor-1 receptor signalling or NF-κB signalling (Engelman and Settleman 2008; Guix et al. 2008; Sequist et al. 2011).

The most frequently occurring secondary EGFR mutation in lung cancer is an exchange of a threonine for a methionine in position 790 (T790M) in exon 20; it is present in about 50 % of all cancers with acquired resistance to EGFR-TKIs (Engelman and Settleman 2008) but very rare in untreated NSCLC (Chmielecki et al. 2012). Additional mutations known to be resistant to gefitinib and erlotinib have also been described and are largely confined to exon 19 and 20, e.g. D761Y, L747S or T854A, and remain apparently rare (Nguyen et al. 2009; Yasuda et al. 2012). Thus, preventing or overcoming ErbB receptor inhibitor resistance with a new generation of ErbB inhibitors promises to increase the durability of treatment. Compounds in clinical development are dacomitinib, neratinib, HM781-36B, AZ 9192 and CO-1686.

**Fig. 1:**
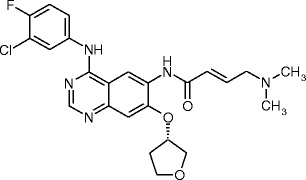
Afatinib

Afatinib (Giotrif®), also known as BIBW 2992 (*N*-[4-[(3-chloro-4-fluorophenyl)amino]-7-[[(3*S*)-tetrahydro-3-furanyl]oxy]-6-quinazolinyl]-4-(dimethylamino)-2-butenamide; Fig. 1), is an ATP-competitive anilinoquinazoline derivative harbouring a reactive acrylamide group, capable of covalent binding to and irreversible inhibition of EGFR, HER2 and HER4 (Solca et al. 2012). It has recently been approved in several countries for the treatment of a distinct type of EGFR-mutated non-small cell lung cancer (NSCLC). This manuscript comprehensively reviews the available non-clinical data on afatinib, whereas clinical data in the treatment of NSCLC (Nelson et al. 2013) and other cancer types (Harbeck et al. 2014) have recently been summarized elsewhere.

## Effects at the molecular level

To explore the direct molecular interaction between ErbB family members and their inhibitors, two types of approach have been used. The most direct one is based on generating crystal structures of the receptor in an inhibitor-bound state (Solca et al. 2012). A more indirect approach uses information from the former as well as data on functional potency of various inhibitors to perform computer-based modelling of the interaction, also known as docking studies, which can be reasonably predictive (La Motta et al. 2009). A docking study of the T790M resistance mutation of the EGFR reported that afatinib binds to a pocket in the catalytic domain including the amino acids Met766, Phe856 and Met790 (Nie et al. 2012). More evidence comes from the analysis of the crystal structure of the afatinib/EGFR kinase domain complex obtained at a 2.8-Å resolution (Solca et al. 2012). This revealed a hydrogen bond with Met793 and a covalent bond between Cys797 and the Michael-acceptor group of afatinib. Covalent binding typically leads to irreversible inhibition, as confirmed with afatinib in intact cells (see next section).

To further explore the covalent binding of afatinib to ErbB receptors, wild-type EGFR, the L858R/T790M EGFR, HER2 and HER4 were incubated with afatinib and digested with pepsin; the resulting digests were separated by liquid chromatography and analysed by mass spectrometry (Solca et al. 2012). These data confirmed the covalent binding of afatinib to EGFR at Cys797 and indicated covalent binding to the corresponding Cys805 and Cys803 in HER2 and ErbB4, respectively.

The functional consequences of afatinib binding to ErbB Family receptors have been studied at the molecular level by measuring inhibition of tyrosine kinase activity in cell-free assays. In such experiments, afatinib showed nanomolar potency to inhibit EGFR, HER2 and ErbB4 (ErbB3 was not studied as it has much weaker tyrosine kinase activity); at each of those receptors, the potency of afatinib was similar to or greater than that of canertinib, erlotinib, gefitinib and lapatinib (Table 1) (Li et al. 2008; Solca et al. 2012). The high potency of afatinib to inhibit EGFR, HER2 and ErbB4 and superiority to gefitinib was confirmed by other investigators (Nam et al. 2011; Sos et al. 2010).

The high potency of afatinib to inhibit the tyrosine kinase activity of wild-type EGFR was fully maintained on EGFR with the activating L858R mutation (Solca et al. 2012; Sos et al. 2010). The potency of afatinib on the L858R/T790M double mutation of EGFR, known to be resistant to erlotinib, gefitinib and lapatinib, was reduced but still in the nanomolar range (Table 1) (Cha et al. 2012; Solca et al. 2012; Sos et al. 2010). When tested in a panel of 52 other tyrosine and serine/threonine kinases, afatinib only inhibited the kinase activity of ErbB family members (Li et al. 2008). The selectivity of afatinib was confirmed by independent investigators assessing the binding properties of afatinib in 442 kinases covering more than 80 % of the human kinome (Davis et al. 2011): it interacted significantly (low nanomolar range; 0.25 nM < *K*_d_ < 6.3 nM) only with EGFR, HER2 and ErbB4; except for GAK, BLK, IRAK1, EpHA6, HIPK4, PhKG2 and phosphorylated Abl-1 kinase (79 nM < *K*_d_ < 570 nM), all other *K*_d_ values were above 1,000 nM, illustrating the high selectivity of afatinib on the human kinome.

## Effects at the cellular level

Studies at the cellular level have been performed to explore two main questions:

- How does afatinib affect intracellular signal transduction in cancer cells?
- What are the effects of afatinib on cancer cell growth and apoptosis?

### Effects on signal transduction

The covalent modification of the EGFR, HER2 and ErbB4 kinase domain by afatinib indicates irreversible inhibition of their enzymatic activity. For EGFR, this was directly confirmed by experiments demonstrating that kinase activity remained inhibited for several hours following afatinib treatment of cell lines for a short time and subsequent washout (Solca et al. 2012). The irreversibility of afatinib effects was independently confirmed for both EGFR and HER2 based on autophosphorylation experiments (Cha et al. 2012).

**Fig. 2:**
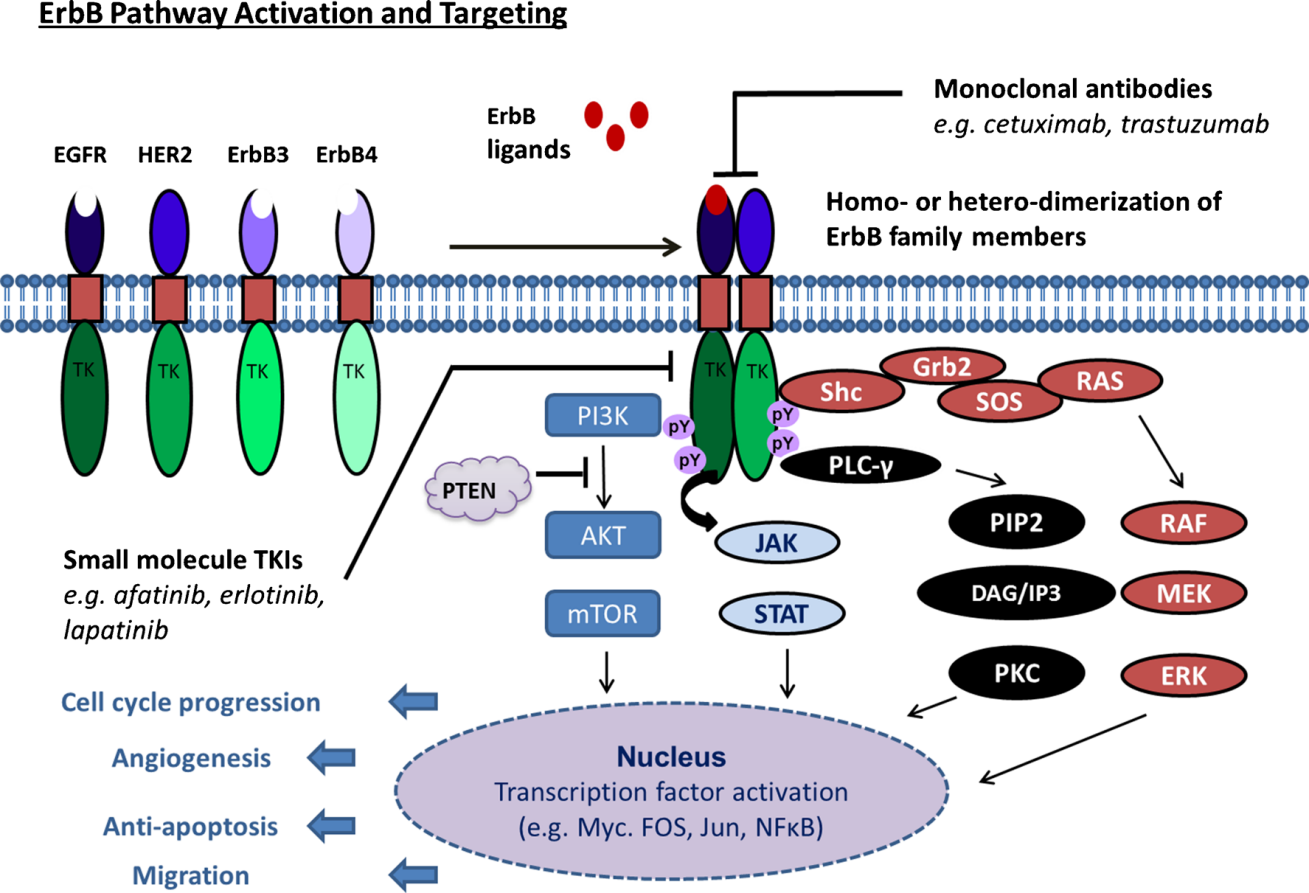
Schematic representation of activation, signalling and targeting of ErbB receptor family members. Overexpression or mutation of ErbB family members (EGFR, HER2, ErbB3 or ErbB4) or overexpression/translocation of ErbB growth factors results in the formation of ErbB homo- and heterodimers; for simplicity only, one heterodimer (EGFR:HER2) is shown here but all homo- and heterodimer combinations between the four monomers are possible. Dimerization leads to the formation of asymmetric head-to-tail complexes of the intracellular kinase domains resulting in transphosphorylation and receptor dimer activation resulting in downstream signalling. ErbB pathway engagement increases cell proliferation, angiogenesis, migration, metastasis and invasion, reduction of apoptosis and/or resistance to radiation and chemotherapy. Anti-ErbB monoclonal antibodies can be directed against EGFR (e.g. cetuximab and panitumab), HER2 (e.g. pertuzumab and trastuzumab) or ErbB3 (e.g. MM121). Small molecule TKIs can be EGFR-specific (e.g. erlotinib and gefitinib), dual EGFR/HER2 (e.g. lapatinib) or pan-ErbB blockers (e.g. afatinib and canertinib)

The first step in the activation of ErbB receptors is ligand-induced dimerization leading to ErbB receptor homo- or heterodimers and subsequent transphosphorylation of tyrosine residues in the C-terminus of the donor kinase domain by the acceptor kinase (Fig. 2) (Zhang et al. 2006). Inhibition of autophosphorylation by afatinib has been shown for EGFR (Ioannou et al. 2013; Kim et al. 2012b; Ninomiya et al. 2013; Solca et al. 2012; Takezawa et al. 2012; Tanaka et al. 2012), HER2 (Canonici et al. 2013; Ninomiya et al. 2013; Solca et al. 2012; Tabara et al. 2012; Takezawa et al. 2012; Tanaka et al. 2012), ErbB4 (Solca et al. 2012) and transphosphorylation of ErbB3 (Ioannou et al. 2013; Kim et al. 2012b; Tabara et al. 2012; Takezawa et al. 2012; Tanaka et al. 2012) in a wide variety of cell lines representing lung (Kim et al. 2012b; Lee et al. 2013; Ninomiya et al. 2013; Solca et al. 2012; Tabara et al. 2012; Takezawa et al. 2012), breast (Solca et al. 2012), gastric (Tanaka et al. 2012), colorectal (Khelwatty SA, Essapen S, Seddon A, Modjtahedi H, in preparation) and pancreatic cancer (Ioannou et al. 2013). Similar to the kinase inhibition studies, afatinib’s potency for inhibition of autophosphorylation was in the low nanomolar range. Moreover, inhibition of autophosphorylation was demonstrated not only in cells expressing wild-type EGFR but also in those containing EGFR mutations including L858R (Li et al. 2008), T790M (Lee et al. 2013; Takezawa et al. 2012) and L858R/T790M (Lee et al. 2013; Li et al. 2008; Solca et al. 2012). Moreover, afatinib inhibits not only the spontaneous or EGF-induced autophosphorylation of EGFR but also that stimulated by TGF-α (Lee et al. 2013). Inhibition of EGFR phosphorylation was also shown for the afatinib/nintedanib combination in a colorectal cancer cell line (Poindessous et al. 2011). Moreover, inhibition of EGFR and HER2 phosphorylation was also demonstrated upon in vivo treatment with afatinib in mice harbouring an adenosquamous lung carcinoma, and this inhibition was further enhanced in combination with rapamycin (Li et al. 2008; Perera et al. 2009).

The inhibitory effects of afatinib have also been explored at the next level of ErbB receptor signalling, i.e. inhibition of downstream signalling pathways such as the Ras/ERK, PI3K/Akt and STAT pathways. Inhibition of ERK activation was reported in cell lines derived from cancers of the lung (Kim et al. 2012b; Lee et al. 2013; Mack et al. 2013; Ninomiya et al. 2013; Tabara et al. 2012; Takezawa et al. 2012), breast (Canonici et al. 2013), stomach (Tanaka et al. 2012) and pancreas (Ioannou et al. 2013). Inhibition of Akt activation was reported in cell lines derived from cancers of the lung (Kim et al. 2012b; Lee et al. 2013; Mack et al. 2013; Ninomiya et al. 2013; Tabara et al. 2012; Takezawa et al. 2012), stomach (Tanaka et al. 2012) and pancreas (Ioannou et al. 2013). Where concentration-response curves were reported, pathway inhibition was observed at low nanomolar concentrations. Such inhibition was additive or even synergistic with that by cetuximab (Takezawa et al. 2012) or by inhibitors of insulin-like growth factor-1 (Ioannou et al. 2013). Inhibition of signal transduction by afatinib was also reported in cells harbouring the EGFR T790M mutation (Kim et al. 2012b; Lee et al. 2013; Takezawa et al. 2012) or cells resistant to gefitinib (Ninomiya et al. 2013) or erlotinib (Mack et al. 2013; Tabara et al. 2012). Afatinib also inhibited activation of additional signal transduction molecules such as the protein kinases p38, rsk1 and p70S6 (Mack et al. 2013). The relevance of the in vitro studies on ERK, Akt and p70S6 was confirmed in vivo in mice harbouring an adenosquamous lung carcinoma, where the inhibitory effects of afatinib were further enhanced in combination with rapamycin (Perera et al. 2009).

In conclusion, these data demonstrate that afatinib irreversibly inhibits important steps in the signal transduction of all members of the ErbB receptor family, which include inhibition of phosphorylation of the receptors themselves and that of downstream signal transduction molecules such as ERK and Akt.

### Effects on cellular growth and apoptosis

The ability of afatinib to inhibit the growth of cancer cells in vitro has been studied in a broad panel of cell lines representing various types of cancer (effects on cell lines harbouring ErbB receptor resistance mutations or being phenotypically resistant to other anti-cancer agents will be covered in the next section). The most frequently studied cancer type is NSCLC, followed by BC and HNSCC.

The first report on afatinib in cellular models of lung cancer initially assessed the effects in two mechanism-based models exclusively driven by oncogenic EGFR mutations, namely, anchorage-independent proliferation of NIH-3 T3 murine fibroblast cells expressing mutant EGFR and IL-3 independent proliferation of the Ba/F3 murine pro-B cell line (Li et al. 2008). Afatinib inhibited colony formation of EGFR-transfected NIH-3 T3 cells in soft agar and was cytotoxic for Ba/F3 cells, the latter confirmed in an independent study (Greulich et al. 2012). It also impaired cell survival in several human NSCLC cell lines, and in each of them, afatinib was more potent than the reversible inhibitors erlotinib, gefitinib and lapatinib or the irreversible inhibitor canertinib. Many subsequent studies have confirmed the growth-inhibiting and cytotoxic properties of afatinib in a wide range of NSCLC cell lines (Cha et al. 2012; Chang and Wang 2012; Greulich et al. 2012; Kim et al. 2012a, b; Köhler et al. 2012; Lee et al. 2013; Nanjo et al. 2012; Ninomiya et al. 2013; Pfeifer et al. 2010; Rho et al. 2011; Solca et al. 2012; Takezawa et al. 2010; Wang et al. 2012). The greater potency of afatinib compared to erlotinib, gefitinib, canertinib or lapatinib was also confirmed in later studies (Ninomiya et al. 2013; Solca et al. 2012; Takezawa et al. 2010). The cytotoxic effects of afatinib were selective for cancer cells with ErbB dysregulation (EC_50_ 1.2–60 nM) as compared to non-malignant cell lines such as Hs-27 human and Balb/c3T3 mouse fibroblasts (EC_50_ 2,835 and 2,105 nM, respectively) (Cha et al. 2012). Of note, not all NSCLC cell lines are equally sensitive to growth inhibition by afatinib. In a study of 65 NSCLC cell lines, the growth inhibitory potency of afatinib varied markedly; it was most potent in cell lines harbouring overexpressed wild-type, mutated or amplified EGFR or HER2 and much less potent in those expressing normal levels of EGFR or mutated BRAF, KRAS or NRAS (Pfeifer et al. 2010). The lower potency of afatinib in NSCLC lines harbouring mutated KRAS was also observed by other investigators (Cha et al. 2012), while other studies revealed that the presence of mutated KRAS had only a little effect on the potency of afatinib in inhibiting proliferation and activate caspase-7, a marker of apoptosis induction (Chen et al. 2012). Because BRAF, KRAS or NRAS mutations have been reported to be non-overlapping with EGFR or HER2 mutations, these data strongly suggest that afatinib behaves like a targeted agent that is selective for cell lines with specific ErbB receptor deregulations and does not affect other cancer cell lines, unless very high concentrations are used.

A role for ErbB targeting agents in the treatment of BC has been well established based on the anti-HER2 monoclonal antibody trastuzumab. The inhibitory activity of afatinib in BC cell lines was explored on 49 BC-derived and five non-malignant breast cell lines (Heiser et al. 2012). The sensitivity to afatinib varied markedly between lines and was about 100-fold greater in lines with amplified HER2 expression in comparison to those with normal expression. Growth inhibition of BC cell lines was confirmed in other studies, where afatinib was consistently more potent than erlotinib, gefitinib, canertinib or lapatinib (Canonici et al. 2013; Cha et al. 2012; Solca et al. 2012). Responsiveness of BC cell lines to afatinib and other HER2 targeting agents was correlated to a specific pattern of gene expression alteration in response to short-term drug exposure (O’Neill et al. 2013). If confirmed, such a functional signature could help in guiding a more specific use of these agents in breast cancer treatment.

Other studies have explored afatinib in HNSCC cell lines. In FaDu cells, expressing EGFR, HER2 and ErbB3, afatinib inhibited proliferation. An increase in the fraction of cells in the G0/G1 stage of the cell cycle with a concomitant reduction of the fraction in the S and G2/M phase was observed and demonstrated the induction of a G1 arrest by afatinib; a small but statistically significant radiosensitizing effect was also noticed in that study (Schütze et al. 2007). Interestingly, pelitinib (EKB-569) has also shown radiosensitizing in vitro effect in SCC-4 cells, which were apparently mediated by inhibition of the binding activity of the transcription factor NF-κB. Inhibition of the NF-κB DNA-binding activity was also dose-dependently modulated by afatinib and neratinib (Aravindan et al. 2011). Growth inhibition by afatinib was further confirmed in additional HNSCC cell lines, and inhibition of the transcription factor “Epithelial-restricted with serine box” by small interfering RNA (siRNA) or a low molecular weight inhibitor reduced EGFR and HER2 promoter activity and potentiated the effects of afatinib in these cells (Zhang et al. 2013).

Finally, the growth inhibitory effects of afatinib have been explored in cell lines representing other types of cancer. For instance, in a study of 11 gastric cancer lines, only two were sensitive to afatinib and other ErbB TKI, and these were the only two that exhibited a high level of HER2 expression (Nam et al. 2012a). Afatinib was confirmed as a potent inhibitor of HER2 overexpressing gastric cancer cell lines in other studies (Cha et al. 2012; Tanaka et al. 2012). In a panel of eight biliary tract cancer cell lines, afatinib exhibited proliferation in low nanomolar concentrations (Nam et al. 2012b). In two of these lines, expressing wild-type KRAS, gefitinib and lapatinib were requiring somewhat higher concentrations; in two other lines expressing mutated KRAS, afatinib retained its potency, whereas gefitinib and lapatinib became ineffective. Afatinib also inhibited growth of nine colorectal cancer cell lines, but its potency varied considerably across lines and was tightly correlated with the expression levels of EGFR, HER2 and ErbB3 (Khelwatty et al. 2011). Growth inhibition by afatinib was also observed in a panel of pancreatic cancer cell lines (Ioannou et al. 2011, 2013). While being tested on a smaller number of cell lines, afatinib exhibited growth inhibition in lines representing bladder cancer (Greulich et al. 2012; Quesnelle and Grandis 2011; Tasi et al. 2012), endometrial cancer (Greulich et al. 2012), basal cell carcinoma (Eberl et al. 2012) and epidermal cell carcinoma (Cha et al. 2012). In melanoma cells with RAS or BRAF mutations, afatinib was found to be ineffective but gained efficacy when used in combination with other drugs (see below) (Held et al. 2013).

## Effects in in vivo model systems

The effects of afatinib in in vivo models have been extensively investigated using human tumour xenograft in immunodeficient mice as well as several transgenic mouse models. Many of these studies were enhanced by ex vivo biomarker analysis on tissue from tumours developed in such mice. The first demonstration of in vivo effects of afatinib was based on xenografts of the epidermoid carcinoma cell line A431; this model expresses high levels of wild-type EGFR and detectable levels of HER2 and had previously been shown to be sensitive to EGFR-targeted antibody treatment (Li et al. 2008). In this model, daily oral treatment with afatinib for 25 days reduced tumour growth and induced regressions. On a molecular level, down-regulation of pEGFR and pAkt expression was observed in treated tumours. In this model, afatinib was more effective than gefitinib or lapatinib when given at their respective maximum tolerated doses.

Afatinib-induced tumour shrinkage was also observed in transgenic mice with inducible expression of the oncogenic EGFR L858R mutant in lung epithelium (Regales et al. 2009); within days, afatinib reduced lung cancer size by more than 80 % as assessed by imaging studies. In another study using constitutive transgenic expression of EGFR L858R in mouse lung, afatinib treatment between weeks 11 and 15 completely prevented the development of lung cancer (Ninomiya et al. 2013). A similar prevention was found in mice with transgenic expression of the deletion 19 EGFR mutant (delE748-A752) upon a 4-week treatment. Long-term treatment with afatinib extended median survival from 119 to 456 days (Ninomiya et al. 2013). Most importantly, tumour burden control by afatinib which resulted in extended survival was not only observed in comparison to vehicle but to gefitinib (Ninomiya et al. 2013). In transgenic mice with inducible expression of mutated HER2 in lung epithelium, afatinib also prevented further tumour growth (Perera et al. 2009). In line with the mode of action of afatinib, this was accompanied by decreased phosphorylation levels of HER2 and various signalling molecules from the ERK and Akt/mammalian target of rapamycin (mTOR) pathway, reduced cellular proliferation (Ki-67) and increased apoptosis.

Similar beneficial effects of afatinib on tumour size were observed in xenograft models of HNSCC (Schütze et al. 2007), gastric cancer (Janjigian et al. 2013; Tanaka et al. 2012), pancreatic cancer (Ioannou et al. 2011), basal cell carcinoma (Eberl et al. 2012), colon cancer (Poindessous et al. 2011) and cetuximab-resistant bladder cancer (Quesnelle and Grandis 2011). Several experiments confirmed that treatment with afatinib reduced tumour volume in xenograft models based on a variety of NSCLC cell lines (Cha et al. 2012; Kim et al. 2012b; Mack et al. 2013; Ninomiya et al. 2013; Pan et al. 2012; Perera et al. 2009; Takezawa et al. 2010, 2012; Yang et al. 2012). Taken together, these studies confirmed the promising in vitro effects of afatinib in in vivo models.

## Afatinib and drug resistance

Afatinib like many other ErbB-targeted agents is primarily effective as a monotherapy in tumours with particular abnormalities of the ErbB signalling network. These defects are essentially confined to overexpression or mutation of ErbB ligands or their respective receptors. ErbB receptor overexpression can be achieved in multiple ways including for instance gene amplification, epigenetic regulation of gene expression or defective protein down-regulation. Downstream molecular defects such as KRAS, BRAF or PI3K mutations as expected do not confer particular sensitivity to ErbB targeting agents. The next sections will focus on treatment resistance mechanisms in NSCLC patients, an indication in which EGFR-TKIs are approved.

### Primary resistance to EGFR-TKIs

Despite the presence of unequivocal ErbB aberrations, primary resistance—which can be defined as resistance to a drug in the case of expected efficacy in patients without prior exposure to this drug—occurs in the clinic. Indeed, not all patients with HER2 amplification respond to trastuzumab; similarly, the radiographic response rate in NSCLC patients with EGFR mutations is generally around 70 %. There are mainly two mechanisms of de novo resistance that have been described so far in NSCLC: The first one involves drug resistant EGFR mutations (e.g. exon 20 mutants or rare mutations such as D761Y or T790M occurring in cis with an activating EGFR mutation), and the second includes other genetic alternations that co-occur with an EGFR-activating mutation in NSCLC tumours. In the preclinical setting, this is well exemplified by the inactivity of EGFR-TKIs in H1650 cells or tumours (NSCLC) that harbour a Del 19 EGFR mutation and are at the same time PTEN-mutated (Sos et al. 2009). Because this defect results in hyper-activation of PI3K signalling downstream of ErbB receptors, it is not surprising that this mechanism contributes to inefficacy of EGFR targeting drugs, including afatinib, despite the presence of an activating EGFR mutation. While many other mechanisms for primary resistance to EGFR-TKI or ErbB targeting monoclonal antibodies are plausible (e.g. expression of multidrug resistance channels, particular metabolizing enzymes, inactivation of tumour suppressors such as PTEN), none of them has been experimentally validated or confirmed in preclinical models or in clinical samples.

### Secondary resistance to EGFR-TKIs

Secondary resistance is defined by the emergence of resistant tumour clones under active treatment. A wide spectrum of molecular abnormalities can contribute to acquired or secondary resistance to ErbB targeting agents in NSCLC (Chen 2012; Sequist et al. 2011).

The first discovered and most frequently observed cause of acquired resistance to gefitinib/erlotinib treatment in NSCLC patients with L858R or Del 19 mutations (50 % of all cases) is the emergence of clones carrying additional EGFR mutations involving the substitution of a threonine residue by a methionine in position 790 (T790M) (Engelman and Settleman 2008). Early experiments suggested that the T790M mutation sterically hindered the binding of TKIs to the EGFR kinase domain (Kobayashi et al. 2005; Pao et al. 2005). Later studies (Yun et al. 2008) revoked the concept and showed that gefitinib actually binds more tightly (5-fold) to the T790M mutant than to EGFR wild type. Surprisingly, this mutant shows an even greater binding affinity for ATP, and the authors suggested that resistance to gefitinib is not attributable to steric blocking but rather to the increased affinity for ATP. The structure of the T790M mutant also suggests that it may accommodate EGFR-TKI such as AEE788 or HKI-272 in different ways. Therefore, several studies have assessed the effect of T790M, studied alone or in combination with the activating mutant L858R, on sensitivity to afatinib. In the first of such studies, afatinib was somewhat less potent as an inhibitor of in vitro tyrosine kinase activity for the L858R/T790M double mutation as compared to the L858R single mutation or the wild-type EGFR; while the double mutation lowered the potency of gefitinib and lapatinib to the micromolar range, that of afatinib stayed in the low nanomolar range (Li et al. 2008); such data were confirmed in later studies (Table 1) (Solca et al. 2012). Afatinib also inhibited phosphorylation of EGFR, ERK and Akt in NSCLC cells with the T790M mutation (Kim et al. 2012a; Köhler et al. 2012; Lee et al. 2013; Ninomiya et al. 2013; Solca et al. 2012; Takezawa et al. 2010, 2012).

Afatinib, but not erlotinib, also inhibited colony formation of NIH-3 T3 cells ectopically expressing several EGFR mutations including L858R/T790M (Li et al. 2008). In models more closely reflecting lung cancer, afatinib also inhibited survival of NSCLC cell lines expressing L858R/T790M (NCI-H1975) or EGFR E746_A750del (HCC827); while the latter was also sensitive to erlotinib, the former was erlotinib-resistant (Li et al. 2008). Growth inhibition of NSCLC lines harbouring EGFR T790M was also reported by other investigators (Chang and Wang 2012; Ercan et al. 2012; Ninomiya et al. 2013; Pfeifer et al. 2010; Sos et al. 2010; Takezawa et al. 2010; Wang et al. 2012). As in the biochemical studies, the T790M mutation reduced the potency of afatinib, but it remained effective within the range which can be achieved by clinically used doses. The inhibitory effects of afatinib have also been reported in many other EGFR genotypes (Sos et al. 2010).

Afatinib inhibited tumour growth in mouse xenograft models harbouring NSCLC cell lines with the T790M mutation; however, the inhibition was only partial and transient (Li et al. 2008; Ninomiya et al. 2013; Pan et al. 2012; Takezawa et al. 2012). Besides xenograft experiments, one in vivo approach to test EGFR mutations consists in using transgenic mice in which lung cancer development is driven by inducible expression of the mutant protein. In such models, afatinib was reported to reduce tumour volume by about half in mice with L858R/T790M-driven lung cancer (Li et al. 2008). Other investigators applied this approach independently to induce lung expression of L858R, T790M and L858R/T790M. In these studies, the L858R genotype was sensitive and the T790M-expressing genotypes insensitive to erlotinib (Regales et al. 2009). While four out of four mice with the L858R genotype exhibited a complete response to afatinib, six out of seven with the double-mutated EGFR exhibited minor tumour regressions (stable disease) and one progressive disease. Modest activity of afatinib was confirmed in the clinic (Sun et al. 2013), indicating the limitation of afatinib monotherapy in this setting.

While T790M is the most frequent EGFR mutation causing acquired resistance to reversible ErbB TKIs, other mutations known to be resistant to these inhibitors have also been studied. EGFR L718Q and L844V were identified as mutations which are not leading to intrinsic activation of the receptor but require EGF for driving proliferation and survival; cells expressing either of these two mutants were sensitive to afatinib (Ercan et al. 2012). Similarly, acquired resistance to EGFR-TKI associated with the second site exon 21 mutation, T854A can be overcome by afatinib in L858R + T854A model systems (Bean et al. 2008).

Loss of addiction to mutant EGFR can also result from a mere loss of the mutation with simultaneous gain of dependence on other ErbB receptors, a process termed “HER reprogramming”. Reprogramming occurs in erlotinib-resistant PC9 clones, and cell survival in the presence of erlotinib is achieved by reactivation of PI3K/AKT signalling through a shift towards HER2/HER3 heterodimers. In these clones, constitutive PI3K/AKT activation was effectively inhibited by lapatinib (EGFR and HER2 inhibitor) and by afatinib (EGFR, HER2 and HER4) as well as by the combination of erlotinib with small interfering RNAs targeting HER2 or HER3 (Tabara et al. 2012). In that study, at least 2 out of 11 patients with activating EGFR mutations who had become refractory to gefitinib treatment displayed EGFR mutation loss at recurrence in disseminated cancer cells from pleural effusions. This data suggests that HER reprogramming could be of relevance in tumours from gefitinib refractory NSCLC patients. The clinical relevance of this resistance mechanism is further underlined by the observation that HER2 is up-regulated in 12 % of erlotinib/gefitinib failures lacking the EGFR mutation T790M (Takezawa et al. 2012). This may in part explain the activity of the afatinib/cetuximab combination (see below).

Besides second site mutations and HER reprogramming, several other mechanisms of secondary resistance to reversible EGFR-TKI have been described in the literature (Chen 2012; Hammerman et al. 2009; Kanda et al. 2013; Lin and Bivona 2012; Sequist et al. 2011). These include (1) MET receptor amplifications (Nanjo et al. 2012), (2) PI3K CA mutations, (3) EMT (epithelial mesenchymal transformation) (Hsu et al. 2012), (4) histological SCLC transformation, (5) activation of AXL, (6) activation of IGF1R-signalling, (7) loss of PTEN and enhanced expression (Sos et al. 2009) and activation of (8) integrinβ1 (Kanda et al. 2013) and (9) SRC (Kanda et al. 2013).

As these resistance mechanisms are not directly related to ErbB receptor aberrations, monotherapy with a newer generation of ErbB targeting agents such as afatinib will not suffice to overcome resistance and drug combinations will be necessary. Selected opportunities are described in a separate section.

### Resistance to afatinib

While several EGFR-mutated cell lines resistant to erlotinib or gefitinib retain sensitivity to afatinib (see above), prolonged exposure to afatinib can also cause resistance. In one study, afatinib-resistant PC9 subclones acquired resistance through T790M mutations with associated changes in the copy number of the EGFR T790M allele. In vitro, these resistant cell lines remained EGFR-dependent as demonstrated by siRNA-mediated down-regulation of EGFR. The xenografts generated from these afatinib-resistant clones were also insensitive to afatinib in vivo and represent a model system in which new treatment options can be tested (Kim et al. 2012b). These data also suggested that gene dosage of EGFR T790M is an important determinant of resistance to afatinib. Of note, amplification of EGFR T790M was also identified as a mechanism of resistance to dacomitinib (Ercan et al. 2010). Both inhibitors are covalent binders and the first step, involving binding to the catalytic cleft of the target prior to covalent engagement to the cysteine, remains necessary. While both compounds differ from reversible inhibitors and show greater inhibitory potency—as ATP competitors—they will face the increased affinity for ATP of T790M EGFR mutants albeit at a much lesser extent. Therefore, tumour expression levels of the T790M mutant protein will impact on efficacy of afatinib and dacomitinib.

**Fig. 3:**
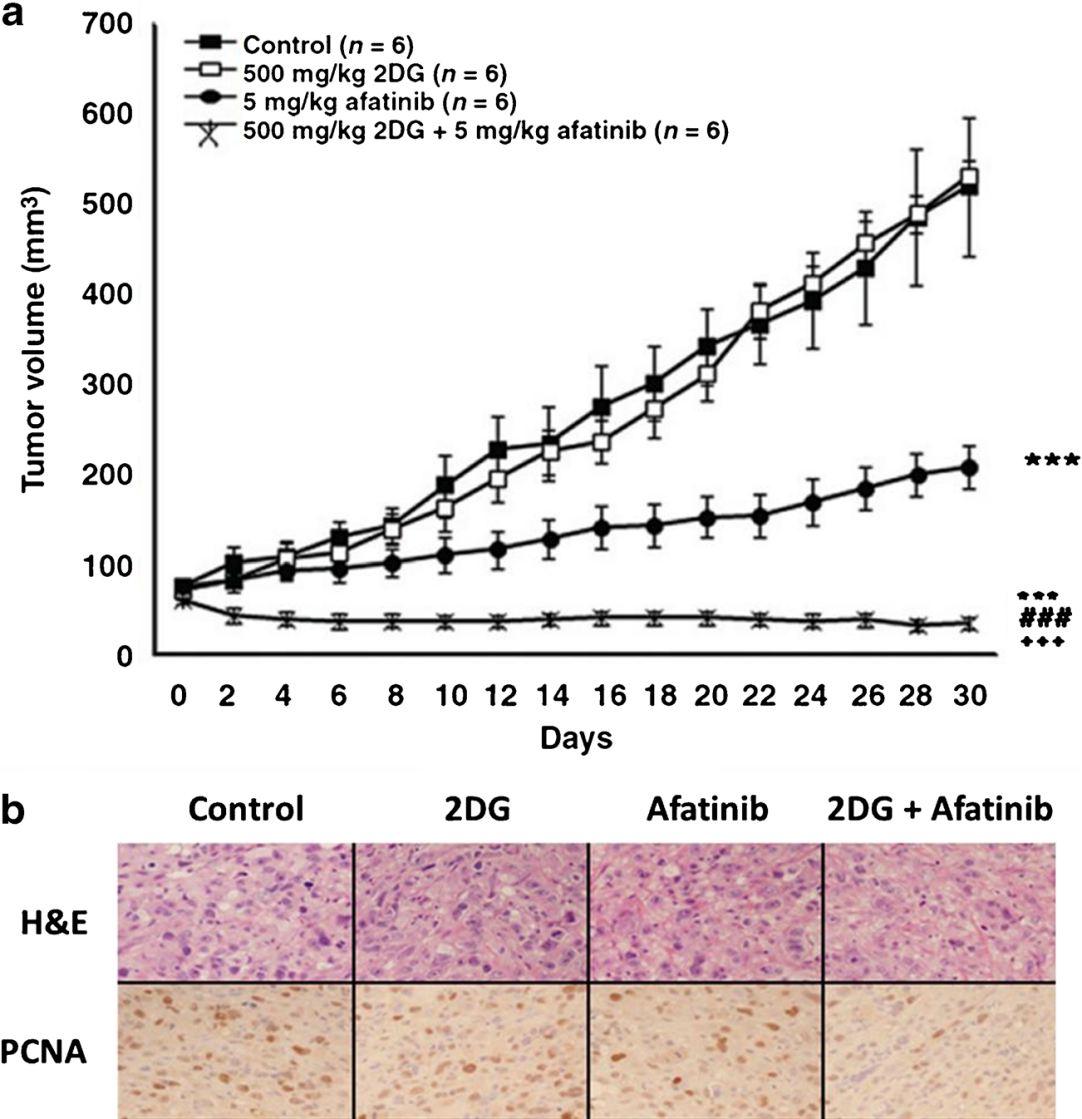
Anti-tumour activity of the glycolysis inhibitor 2-deoxy-d-glucose (2DG), afatinib and their combination as compared to control (CON) in a PC9-GR tumour xenograft model. *Upper panel*: Mice bearing PC9-GR xenografts received the indicated drugs daily. Data represent mean ± SE. ***P < 0.001 vs. control; ###*P* < 0.001 vs. 2DG; +++*P* < 0.001 vs. afatinib. *Lower panel*: After 5 days of drug treatment, mice were sacrificed and tumour tissue was analysed by histology (haematoxylin/eosin staining) and immunohistochemistry for the proliferation marker PCNA. Reproduced from Kim et al. (2013)

In a second study assessing resistance to afatinib in T790M containing mutants, it was found that administration of the drug resulted in an increase in mRNA coding for the cytokine IL-6 (Kim et al. 2012a, b). In this case, autocrine IL-6 secretion was shown to activate the JAK1/STAT3 pathway (increased pSTAT3 signal in afatinib treated H1975 or PC9-GR cells), and inhibition of IL-6 or JAK1 restored afatinib sensitivity in vitro. The combination of afatinib and P6, a pan JAK inhibitor, was tested in vivo in the gefitinib-resistant PC9-GR NSCLC model which carries a combined Del19 T790M EGFR mutation (Fig. 3). The enhanced anti-tumour effect of the combination was further corroborated by immunohistochemical analyses of PCNA (proliferation marker).

### Afatinib in resistance to chemotherapy

Platinum doublets remain the mainstay of therapy in lung cancer. In the EGFR-mutated population, several trials including IPASS, EURTAC, LUX-Lung 3 and LUX-Lung 6 have clearly demonstrated the superiority of ErbB targeting agents over standard platinum doublets (Langer 2013). Nevertheless, treatment with for instance permetrexed and cisplatinum will remain the main second line treatment in this population. Because EGFR-mutated tumours often remain sensitive to ErbB targeting agents beyond progression, thus allowing re-introduction of these agents in later lines of therapy, it is important to test whether chemotherapy impacts on the sensitivity to these drugs. To this end, cisplatinum-resistant NSCLC cell lines were generated from lines expressing either wild-type (A549 and H460) or mutant EGFR (PC-9 and HCC827) (Rho et al. 2011). Results from investigations assessing proliferation and induction of apoptosis revealed that the potency of erlotinib, gefitinib and afatinib and the apoptotic fractions induced by them were almost identical in cisplatinum-resistant and parental lines. The objective response rate of 61 % observed in LUX-Lung 2 (second line use of afatinib in EGFR mutant NSCLC patients) confirms the observation in the clinical setting. Taken together, these data demonstrate that resistance to classic chemotherapeutic agents does not necessarily cause resistance to ErbB TKIs.

## Afatinib in combination treatments

The use of combination treatments is frequent in oncology and aims at delaying or even preventing the development of resistance. To this end, non-clinical models have been used to explore the potential of afatinib in combination with other drugs. An obvious option is the combination of afatinib with other ErbB inhibitors. The rationale behind this concept is maximization of ErbB pathway silencing. The preferred option has been to use monoclonal antibodies, such as anti-EGFR cetuximab and anti-HER-2 trastuzumab, as combination partner for afatinib taking into account that afatinib blocks the kinase activity of all active ErbB receptor kinases. In a transgenic model with lung-directed expression of EGFR L858/T790M mutation, afatinib showed only moderate efficacy. Minor tumour regressions (some up to 50 %) were observed in this model (Li et al. 2008; Regales et al. 2009); however, the combination of afatinib and cetuximab reduced tumour volume to a much greater extent (eight out of eight tumours beyond 50 % reduction with six out of eight beyond 80 %) than either drug alone (Regales et al. 2009). This combination was also more effective than single agent treatments in the H1975 xenograft model harbouring the L858R/T790M mutation. The combination potential of afatinib with other EGFR targeting antibodies (panitumumab) was confirmed in a second xenograft model (PC9/BR) generated from a different NSCLC cell line with the same EGFR genotype (Takezawa et al. 2012). That study furthermore corroborated the in vivo synergism for both antibodies in in vitro studies. In these experiments, the combination provided greater inhibition of phosphorylation of EGFR, HER2, ErbB3, Erk and Akt than either drug alone.

The afatinib/cetuximab combination may have benefits beyond the T790M mutation. In an independent study, afatinib treatment induced complete tumour response in mice implanted with tumour fragments from a patient with acquired erlotinib resistance (but lacking T790M), but after cessation of treatment, tumours progressed within 2 weeks; however, when the initial treatment consisted of an afatinib/cetuximab combination, the mice remained in complete remission during the entire 75-day follow-up (Mack et al. 2013). Additional observations support the concept of multi-level ErbB receptor inhibition. EGFR knock-down by siRNA enhanced the growth inhibitory effect of afatinib or cetuximab in a panel of five NSCLC cell lines, including those with T790M mutation (Chen et al. 2012). However, in an EGFR-mutated cell line resistant to EGFR inhibition in vitro most likely due to a exon 9 deletion in PTEN (H1650), the combination of afatinib and cetuximab was not effective (Chang and Wang 2012). An EGFR antibody, ICR62, exhibited supra-additive growth inhibition with afatinib in a colorectal cancer cell line (DiFi) (Khelwatty et al. 2011). The combination of afatinib and cetuximab has also been evaluated clinically in patients failing on erlotinib or gefitinib therapy and early results from the phase I trial showing a 40 % response rate are promising (Janjigian et al. 2012). Finally, similar synergism may also apply to HER2 targeting. In a panel of eight BC cell lines, with or without resistance to lapatinib, the combination of afatinib with trastuzumab was more effective than either drug alone; such synergism was also observed in one line from a subset of four lines with trastuzumab resistance (Canonici et al. 2013). The clinical results of a trial assessing the combination of trastuzumab and lapatinib in HER2-positive breast cancer patients also support the combination paradigm of dual targeting of HER2 (Blackwell et al. 2010). All these examples are in concordance with the concept that maximal ErbB pathway inhibition can be achieved with a combination of a monoclonal antibody and a small molecule targeting the same dysfunctional ErbB receptor.

Afatinib was not only tested in combination with inhibitors of ErbB receptors but also with inhibitors targeting specific downstream molecules in the signalling pathway, e.g. MEK, Akt, PI3K, mTOR or STAT3. For instance, in a bladder cancer cell line (5637), harbouring a S310F mutation of HER2, afatinib alone had a little effect on cell survival, but in the presence of an inhibitor of MEK, which did not affect survival on its own, afatinib became a 10-fold more potent inhibitor (Greulich et al. 2012). In an NSCLC model (H1975, EGFR T790M), combinations of afatinib with a MEK inhibitor (PD032901), a SRC inhibitor (dasatinib) or a PI3K inhibitor (PI-103) showed additive effects on induction of apoptosis, although this did not reach statistical significance (Sos et al. 2010). Dasatinib, an inhibitor of BCR-Abl kinase and SRC kinase, exhibited synergistic growth inhibition with afatinib in NSCLC lines expressing L858R/T790M or the combination of del E746-A750 with a deletion of exon 9 in PTEN (Chang and Wang 2012). In BRAF mutant melanoma cell lines, afatinib also had a little effect as a single agent but became a more potent growth inhibitor in the presence of Akt inhibitors (GSK690693 and MK-2206) (Held et al. 2013). Pfeifer and colleagues have systematically explored the compound combination PI-103 (dual PI3K/mTOR inhibitor) with afatinib on a panel of 65 NSCLC cell lines (Pfeifer et al. 2010). The authors identified 11 cell lines for which the combination treatment was synergistic. Amplifications or mutations in either EGFR or ERBB2 were not enriched in this subset of cell lines, suggesting a preferential engagement of PI3K signalling downstream of activated EGFR and HER2. The combination of afatinib with the JAK1 inhibitor P6 was synergistic with regard to apoptosis induction in a NSCLC cell line harbouring EGFR T790M (Kim et al. 2012a; Sos et al. 2010), supporting the finding described in Fig. 3. The role of mTOR was specifically assessed in several studies using the mTORC1 inhibitor rapamycin. This combination was reported to be synergistic for in vivo inhibition of lung cancer growth in a transgenic EGFR L858R/T790M model (Li et al. 2008) and in transgenic as well as xenograft HER2 mutant models (Perera et al. 2009). Inhibition of glycolysis using 2-deoxy-d-glucose sensitized NSCLC cells with EGFR T790M mutation to afatinib via translational suppression of Mcl-1 by activation of AMP-activated protein kinase (Kim et al. 2013). This long list of examples demonstrates that afatinib provides ample opportunities for combinations with targeted agents.

**Fig. 4:**
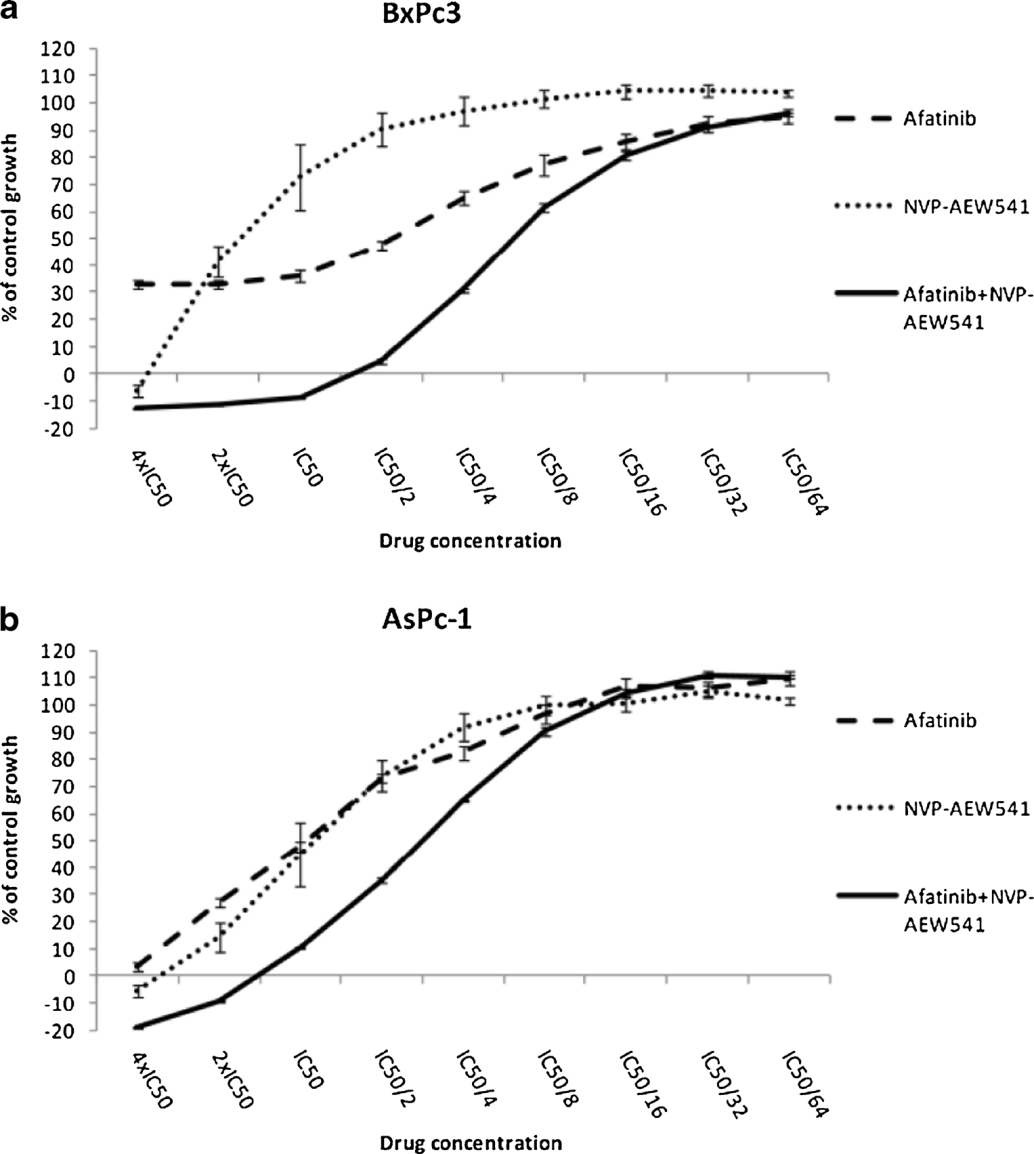
Synergistic growth inhibition of human pancreatic cancer cells following treatment with a combination of afatinib and the IGF-IR inhibitor NVP-AEW541. **a** BxPc3, **b** AsPc-1 cell line. Reproduced from Ioannou et al. (2013)

Finally, afatinib has also been explored in combinations with agents not directly acting on ErbB receptors or their signalling pathway. For example, afatinib in combination with the chemotherapeutic drugs 5-fluorouracil or permetrexed, which target thymidylate synthase, synergistically inhibited proliferation of a gefitinib-resistant, T790M-expressing NSCLC cell line, likely due to afatinib-induced suppression of enzyme expression (Takezawa et al. 2010). In that study, afatinib was also synergistic with permetrexed and with a thymidylate synthase inhibitor in an in vivo xenograft model; in contrast, gefitinib did not exhibit such synergism. In an in vitro study with colorectal cancer cell lines, the combination of afatinib with 5-fluouracil was antagonistic in three lines, but additive or even synergistic in two others (Khelwatty et al. 2011). The combination of afatinib with the VEGF antibody bevacizumab was found to be superior to either drug alone in mice harbouring NSCLC xenografts with an EGFR exon 19 deletion/T790M or with a L858R/T790M mutation (Ninomiya et al. 2013). In a panel of seven pancreas cancer cell lines, afatinib caused synergistic growth inhibition when combined with an insulin-like growth factor receptor antibody (Ioannou et al. 2013) (Fig. 4).

In HNSCC cell lines, inhibition of the transcription factor ESX (epithelial-restricted with serine box) by siRNA or by a low molecular weight inhibitor reduced EGFR and HER2 promoter activity; the growth-inhibiting effects of these approaches were enhanced by afatinib (Zhang et al. 2013). Radiation treatment of bladder cancer cells dose-dependently induced phosphorylation of EGFR, HER2 and Akt; in a colony formation assay, afatinib showed synergism with radiation in such cells, indicating possible radiosensitization (Tasi et al. 2012). A minor radiosensitization by afatinib had also been observed in HNSCC cell lines (Aravindan et al. 2011; Schütze et al. 2007).

## Conclusions

The preclinical data summarized in this review demonstrate that the irreversible pan-ErbB TKI afatinib is effective against a wide range of cancers driven by aberrant ErbB receptor signalling. Afatinib also shows activity against cancers resistant to first-generation EGFR inhibitors due to certain activating EGFR mutations, secondary EGFR mutations such as T790M or tumours expressing other ErbB family receptors activated by drug-induced reprogramming of signalling pathways. Afatinib has recently been approved in several countries for the treatment of patients with a distinct type of EGFR-mutated non-small cell lung cancer. A multitude of clinical trials examining afatinib in lung cancer as well as additional cancer types and in combination with a variety of established or investigational agents is currently on-going; the results of these studies will reveal its full potential to improve treatment outcomes for a large number of cancer patients with abnormalities of the ErbB network.

## Abbreviations

BC: Breast cancer
EGF: Epidermal growth factor
EGFR: Epidermal growth factor receptor
ERK: Extracellular signal-regulated kinase
HNSCC: Head and neck squamous cell cancer
mTOR: Mammalian target of rapamycin
NSCLC: Non-small cell lung cancer
PI3K: Phosphatidyl-inositol-3-kinase
STAT: Signal transducers and activators of transcription
TGF-α: Transforming growth factor-α
TKI: Tyrosine kinase inhibitor

## Footnotes

1 Some investigators use the term EGFR for the entire class of ErbB proteins. In line with most others, we here use it only for ErbB-1/HER1.
